# Nonadaptive Fluctuation in an Adaptive Sensory System: Bacterial Chemoreceptor

**DOI:** 10.1371/journal.pone.0011224

**Published:** 2010-06-29

**Authors:** Masatoshi Nishikawa, Tatsuo Shibata

**Affiliations:** 1 Department of Mathematical and Life Sciences, Hiroshima University, Higashi-Hiroshima, Hiroshima, Japan; 2 CREST, Japan Science and Technology Agency, Suita, Osaka, Japan; 3 PRESTO, Japan Science and Technology Agency, Kawaguchi, Saitama, Japan; Mount Sinai School of Medicine, United States of America

## Abstract

**Background:**

Sensory systems often exhibit an adaptation or desensitization after a transient response, making the system ready to receive a new signal over a wide range of backgrounds. Because of the strong influence of thermal stochastic fluctuations on the biomolecules responsible for the adaptation, such as many membrane receptors and channels, their response is inherently noisy, and the adaptive property is achieved as a statistical average.

**Methodology/Principal Findings:**

Here, we study a simple kinetic model characterizing the essential aspects of these adaptive molecular systems and show theoretically that, while such an adaptive sensory system exhibits a perfect adaptation property on average, its temporal stochastic fluctuations are able to be sensitive to the environmental conditions. Among the adaptive sensory systems, an extensively studied model system is the bacterial receptor responsible for chemotaxis. The model exhibits a nonadaptive fluctuation sensitive to the environmental ligand concentration, while perfect adaptation is achieved on average. Furthermore, we found that such nonadaptive fluctuation makes the bacterial behavior dependent on the environmental chemoattractant concentrations, which enhances the chemotactic performance.

**Conclusions/Significance:**

This result indicates that adaptive sensory systems can make use of such stochastic fluctuation to carry environmental information, which is not possible by means of the average, while keeping responsive to the changing stimulus.

## Introduction

Adaptation is a common mechanism for sensory and regulatory systems to be responsive to a changing stimulus over a wide range of background concentration [1]. When the sensory system is exposed to changes in background stimulus, the system responds by altering its activity, which is then followed by adaptation back to its prestimulus level. This adaptive response is considered to reset the system to be ready for a new signal and prevents saturation of the response. However, because of this adaptation property, the sensory system cannot carry any information about the background. If the system could make use of such information, yet remain responsive to the changing stimulus, it would be advantageous.

**Figure 1:**
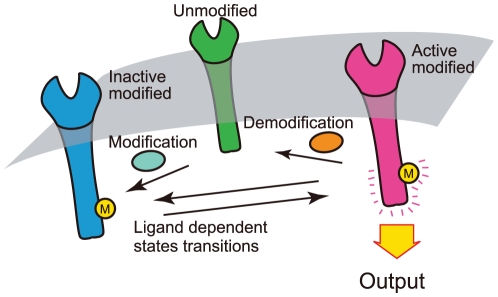
Two-state adaptive sensory model. A schematic of the two-state adaptive sensory model. The signaling molecule in the modified state has active and inactive states. The transition rates between them are dependent on ligand concentration. The activity 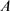 is the number of active molecules, which is the output for downstream systems. If the transition rates between the modified and unmodified states are dependent solely on 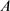, a perfect adaptation is achieved (see text). In the case of a bacterial chemoreceptor, the signaling molecule is a receptor, and the modification and demodification reactions are catalyzed by enzymes CheR and CheB respectively.

A general and simple mechanism to achieve adaptive response is the activity-dependent kinetics, in which a sensory molecule is reversibly modified depending on its activity [2]. When an environmental condition changes, equilibrium between two functional states, active and inactive, immediately shifts to generate a response in the activity of sensory molecules. Then, the modification reaction takes place to counterbalance the change in activity so that it returns to the prestimulus level [3]. When the rates of modification and its reverse reactions depend solely on its activity, the stationary activity level is independent of the environmental conditions and exhibits adaptation (Fig. 1). Bacterial chemotaxis is one such system in which a methylation reaction is responsible for adaptation [2]. When a protein has two conformational states, which are distinguishable from both active and inactive states, with the rate of conformational change depending only on its activity, the activity of the protein exhibits an adaptive response. Some ion channels show such activity, having two functional states, active and inactive, and an additional non-conducting conformational state [4]. The internalization of some receptors, such as G-protein-coupled receptors, is also responsible for the adaptive response of the receptor activity [5].

**Figure 2:**
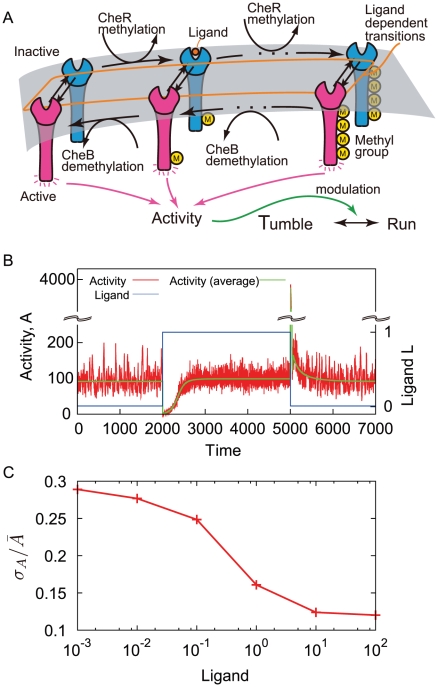
Stochastic property of the two-state bacterial receptor model with the multiple methylation sites. A. Schematic of the two-state bacterial chemoreceptor model. The state transitions between the active and inactive states take place with transition rates which are dependent on the ligand concentration 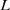. The ratio of the transition rates is also dependent on the methylation level. Inactive receptors are methylated by CheR, whereas active receptors are demethylated by CheB. B. Stochastic response and adaptation of bacterial chemoreceptor activity. The activity 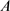 as the number of active chemoreceptors (red) is plotted as a function of time. For step increase and decrease in chemoattractant concentration (blue), the activity shows response and adaptation. All the rate constants used here are the same as in Ref. [24]. To perform a stochastic simulation using Gillespie algorithm [31], we suppose the cell volume to be 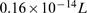 so that 1nM is equivalent to one molecule per cell. C. The dependence of activity fluctuation on the chemoattractant concentration. The relative fluctuation of activity 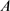 defined as the ratio between the standard deviation and its mean plotted as a function of the chemoattractant concentration 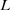.

Among sensory systems, bacterial chemotaxis is an extensively studied system where the adaptive response plays an essential role. The motion of a bacterium consists of a series of “runs”, moving smoothly, interrupted by “tumbles”, changing its direction randomly [6]. For a step increase in chemoattractant concentration, the tumbling frequency exhibits a transient decrease followed by an increase up to the prestimulus level [7], [8]. Such an adaptive response is known to be generated by the bacterial chemoreceptor complex [9]. For a sustained increase of ligand concentration with time, the adaptive system generates a persistent shift of its activity from its adapted level, which suppresses the tumbling so that the bacterium can climb the gradient [10]. In this way, the adaptive response is essential for bacterial chemotaxis. In the biochemical network of bacterial chemotaxis, the methylation and demethylation of chemoreceptors by enzymes CheR and CheB are responsible for the adaptation. With a change of chemoattractant concentration, the tumbling frequency is modulated. The covalent modification compensates for the change in tumbling frequency. A two-state model for the activity-dependent kinetics has been proposed to account for the properties of this adaptive response [2],[11]–[14] (Figs. 1 and 2A).

Such a biochemical computation is operated by the stochastic reactions of biomolecules, which makes signal transduction inherently noisy [15]–[17]. The adaptation may be achieved on average. However, the activity of the chemoreceptor will inevitably exhibit temporal deviations from the adaptation level. Korobkova et al. found that the tumbling frequency exhibited large and relatively slow temporal fluctuations under the no chemoattractant condition and the time duration of counterclockwise rotation of the flagellar motor showed a heavy-tailed distribution away from exponential distribution [18]. Their experimental data suggested that this large behavioral variability was a result of the fluctuation generated in the chemoreceptor adaptive response circuit. Emonet and Cluzel have discussed theoretically the effect of stochastic fluctuation in the chemoreceptor process on the motile behavior of bacteria [19]. They showed that under the absence of chemoattractant, the time constant of the receptor activity increases with the increase in the level of stochastic fluctuations, as in the case of the covalent modification cycle [20]. They further showed that such an increase in the time constant can contribute to increase in the velocity up the chemoattractant gradient.

Since such a stochastic sensory system is working over a wide range of background, the question can be asked whether the fluctuation is an adaptive property in an adaptive sensory system, and whether it can perform any role to sense changes in stimuli. To answer these questions, here we first study a simple prototypical model which unifies many adaptive systems. Based on this model, we show theoretically that the fluctuation can be a nonadaptive property, while the system carries out the adaptation on average. We then perform a numerical simulation on the detailed bacterial chemoreceptor model to verify our theoretical result, which in fact exhibits the nonadaptive fluctuation. Such a property of fluctuation makes the bacterial behavior dependent on the chemoattractant ligand concentration. As a result, the chemotactic performance can be improved.

## Results and Discussion

### A simple two state model of adaptive response

To study the essential properties of fluctuation in an adaptive system and its underlying mechanism, we here study a simple two state model that responds and adapts to a change in the environmental conditions. In the present simplified two-state model, each molecule is in one of the two states, active and inactive, between which transition reactions take place. The rates of the transition reactions are dependent on the environmental ligand concentration. When the environmental ligand concentration is changed, the equilibrium between the two states is broken, leading to a transient increase or decrease in the number of active molecules, 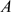. Such cases are often observed in sensory systems, such as receptors, which are activated upon binding or unbinding of ligands. For instance, in the case of bacterial chemotaxis, the activation probability of the chemoreceptor decreases as the increase of chemoattractant concentration. In the case of chemotactic cells *Dictyostelium*, the activation probability of the G-protein coupled receptor cAR1 (cAMP receptor) increases with the cAMP concentration.

Adaptation occurs when modification of molecules can also affect the equilibrium between the active and inactive states. After a transient response to a change in stimulus, the modification or demodification reactions occur, which shift the equilibrium between two states to compensate for the transient response. As a result, the number of active molecules, 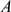, exhibits an adaptation. The adaptation can be perfect, when the rates of modification and demodification reactions are determined by 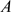 alone. Here, for simplicity, we consider a single modification step. Thus, each molecule is either modified or unmodified. The equilibrium constant between active and inactive states is dependent on these modification states. In the extreme case, the unmodified state, denoted by 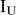, is always inactive. The modified state consists of an active state A and inactive state 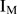, between which transition reactions can take place. There are many possible kinetic schemes for the adaptive response. Here, we consider the following simple kinetic scheme (Fig. 1):
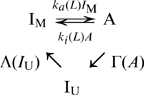
(1)where 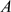, 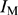, and 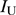 are the number of molecules of A, 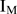 and 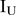, respectively, 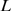 is the ligand concentration, 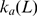 and 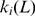 are the rate constants of the activation and deactivation reactions between A and 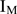 in the modified state, which are dependent on the ligand concentration 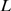, and 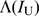 and 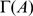 are the rate constants of the modification and demodification reactions, respectively. Here, for the modification and demodification reactions, we consider the enzymatic reaction described by the Michaelis-Menten equation, given by 
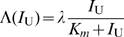
 and 
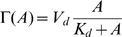
, respectively. We note that this mechanism requires that the system works far from thermodynamic equilibrium.

At steady state, since the modification and demodification reactions are balanced, we obtain 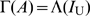 from scheme 1. To achieve a perfect adaptation of activity 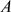, the modification reaction should perform at a saturating level, 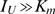, giving 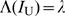 [2]. As a result, the rate of modification and its reverse reactions are dependent solely on the activity 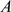. Under such a condition, we obtain the equation 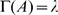 at steady state. Notice that no parameter dependent on 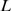 is included, indicating that the steady state level of 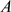 obtained as a solution of this equation is independent of 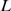 and the model exhibits a perfect adaptation against changes in 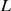.

Since the total concentration is conserved and the modification reactions are working under the saturation condition 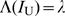, the reaction scheme can be reduced to
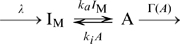
(2)We consider the stochastic kinetics described by the chemical Langevin equation [21], given by
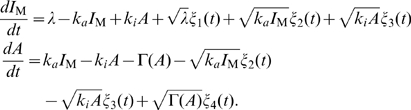
(3)The last three terms in each equation are noise terms because of the stochastic occurrence of the reactions. Here, 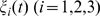 is white Gaussian noise with zero mean and 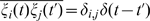.

### Stochastic fluctuations of activity in adaptive systems is not adaptive

To study whether the property of stochastic fluctuation in the activity 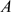 is adaptive or not, we calculate the variance of 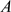 by solving the chemical Langevin equation shown in Eq. (3), adopting the linear noise approximation. In a steady state, the fluctuation intensity 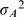 is given approximately by
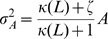
(4)with 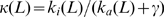, 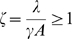, and 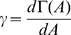 (see Materials and Methods for details). Here, 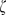 is a constant, and is regarded as a measure of the non-first order (nonlinear) degree of the demodification reaction, given by, 
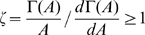
. Thus, from Eq.4 the fluctuation intensity 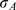 can be dependent on the absolute concentration 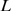 through 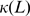, and it is a nonadaptive property of adaptive sensory systems. Since reactions specific to a particular system are restricted to the form of 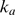 and 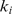, this nonadaptive property in the stochastic fluctuation is considered as a property common to the class of models considered here.

What property of our model makes the fluctuation of activity 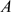 dependent on the ligand concentration 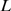
? When the demodification reaction performs as a first order reaction, 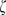 is unity. In such a case, according to Eq. (4), the fluctuation 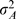 becomes 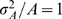 and is independent of 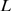. When the inactivation reaction is not present, 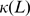 vanishes and the fluctuation 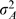 becomes 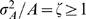, which is also insensitive to 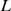. Therefore, to have nonadaptive fluctuation these two depletion pathways for the active form A, both the inactivation and demodification reactions, are necessary, where at least one of them should be a non-first order reaction. As we shall see later, the major reaction to deplete A changes from the demodification reaction to the inactivation one with the increase of 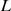.

### Nonadaptive fluctuations in reduced activity dependent kinetics

When the transition between active and inactive states is faster than the modification and demodification reactions as is often supposed, the present model can be further simplified. In the case of a bacterial chemoreceptor, the methylation and demethylation reactions are usually supposed to be much slower than the activation and inactivation reactions. In the case of ion channels, the transition between conducting and non-conducting states involves a conformational change, which is expected to be much slower than the transition between open and closed states. The internalization of receptors is also a slow process compared with the activation and inactivation reactions. In the present model, the modified state M, which consists of active state A and inactive modified state 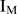, is produced and depleted according to the following reaction scheme,
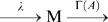
(5)When the state transition between A and 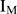 is much faster than the modification and demodification reactions, it can be regarded as in equilibrium. Then the number of A is determined by the reaction,
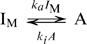
(6)Thus, the number of 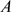 is expected to follow the binomial distribution conditional on a given number of the molecules in modified state M, 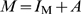. Thus, the present two state model can be described by the chemical Langevin equation for modified level 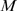, given by
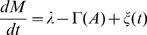
(7)with
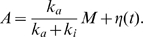
(8)where 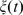 is the Gaussian white noise with 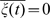 and 
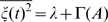
, and 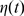 is a random number of the normal distribution with zero mean and the variance given by 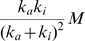. We notice that Eqs.(7) and (8) are considered an extension of the system level approach to an adaptive system proposed in Ref. [22] to include the effect of stochastic fluctuations.

By solving the chemical Langevin equation (7) with the linear noise approximation, we obtain the variance of 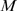 as 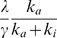 (see Materials and Methods for details). Thus, by adding the variance of the binomial distribution, the variance of the activity 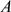, 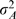, is given by
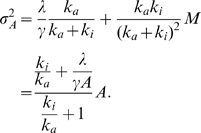
(9)Since 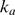 and 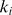 are dependent on the ligand concentration 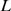, the relative noise intensity 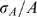 is dependent on the absolute ligand concentration 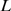, indicating again that it is a nonadaptive property of adaptive sensory systems. We note that the expression in Eq.(9) can be obtained from Eq. (4) by assuming that the activation reaction is much faster than the demodification reaction, i.e., 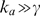.

### Stochastic fluctuation is not an adaptive property of bacterial chemoreceptor system

In the case of bacterial chemoreceptors, the modification and demodification reactions are performed by methyltransferases CheR and CheB, respectively. The unmethylated state is usually assumed to be inactive [23]. For simplicity, we consider first the case with a single methylation step. For the methylated receptors, the forms of activation and inactivation reaction rates 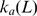 and 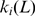 in scheme 2 are chosen so as to satisfy bacterial chemotaxis where the tumbling rate decreases (increases) when the ligand concentration increases (decreases). Thus, 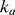 and 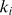 are respectively decreasing and increasing functions of 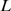. We adopt simplest forms given by 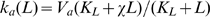, and 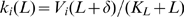, where, 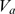 and 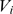 are the maximum velocities, 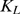 is the dissociation constant of the ligand, and 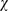 and 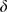 are constants with 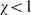 and 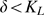. Even under the no chemoattractant condition, the activation and inactivation rates are respectively given by 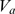 and 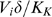, which makes 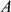 and 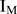 able to reach equilibrium even without the methylation and demethylation reactions. The steady state level of 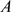 is obtained as 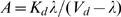, which is independent of 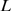 showing a perfect adaptation.

**Figure 3:**
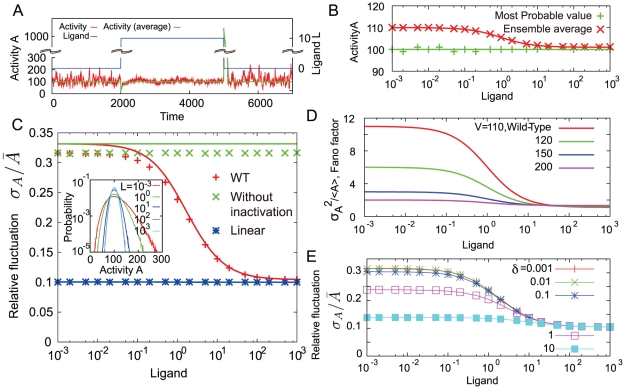
Stochastic property of the two-state bacterial sensory model. A. Stochastic response and adaptation obtained by scheme 2. The activity 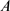 is plotted as a function of time. For step increase and decrease in ligand concentration (blue line), the activity shows the response and adaptation. The time series obtained by the corresponding kinetic equation without the noise term is shown by the green line. B. Adaptation of activity 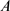. The ensemble average of 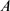, 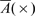, and the most probable value of 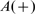, obtained by the stochastic simulation are plotted as functions of the ligand concentration. The activity 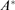 obtained by the kinetic equation without the noise term (green line) is also depicted. The deviation between 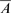 and 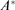 is due to the nonlinearity of the demodification reaction. The theoretical result including the nonlinear effect is also shown (red line, see Materials and Methods). C. The dependence of activity fluctuation on the ligand concentration. The relative fluctuations of activity 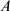, 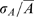, obtained by stochastic simulation are plotted as functions of chemoattractant concentration 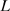 for scheme 2(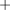), without the inactivation reaction with 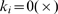, and without the nonlinear effect in which 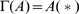. The respective theoretical lines are also plotted. Inset: The distributions of activity 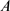 for several ligand concentrations. D. The effect of the activation and inactivation reaction rates, 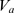 and 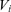, on the nonadaptive fluctuation. The fluctuation strength 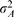 is calculated according to Eq. (4) for each 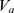 and 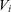. The other parameter values are the same as indicated in Materials and Methods. E. The effect of the ligand independent inactivation reaction, 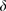, on the nonadaptive fluctuation. For each value of 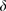 as indicated in the figure, the fluctuation strength 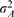 is calculated according to Eq. (4).

We performed a stochastic simulation of scheme 2 for the case of bacterial chemoreceptor as shown in Fig. 3A (see Materials and Methods for the detail of the simulation method). The time course shows that the increase (decrease) of 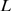 results in a transient decrease (increase) of activity 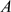 and thus the tumbling frequency. After the transient response, the stochastic time course of 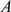 exhibits a perfect adaptation (Fig. 3A) over a range of more than six orders of ligand concentration (Fig. 3B). The most probable value of 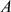 perfectly adapts to the background ligand concentration. The ensemble average of 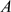, 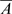, deviates slightly from the stationary value under low background, because the distribution of 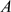 is skewed to the right (Fig. 3C inset). The modification level 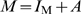 increases with the increase of 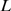, which is consistently observed in the experiments of bacterial chemoreceptor reported previously [23].

Fig. 3C shows the relative fluctuation 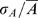, which is not a constant but a decreasing function of 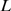. As 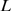 decreases to zero, 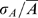 approaches a saturation level, while 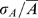 is decreasing to a lower bound level with increasing 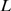. Thus, while the mean level of activity is an adaptive property, the stochastic fluctuation is a nonadaptive property sensitive to the ligand concentration. Therefore, the stochastic activity can still bear information of the chemoattractant ligand concentration.

In Fig. 3C, the theoretical result of stochastic fluctuation in the two state model given by Eq.(4) is applied to the case of bacteria, exhibiting a good agreement with the numerical result. For this case, 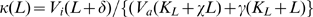 is an increasing function of 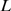. According to Eq. (4), when the ligand concentration 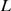 is sufficiently small, 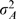 is given by 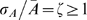. As 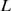 increases, 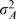 decreases approximately in proportion to 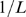. Then, as 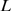 increases further, 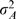 approaches 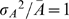.

Such a decrease of the fluctuation 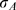 with the increase of 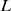 is due to the shift of effective depletion pathway of A from the demodification reaction to the inactivation reaction. As the ligand concentration decreases to 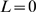, the rate of the inactivation reaction is reduced. Since in such a case the time constant of 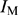, given by 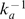, is much smaller than that of 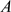, given by 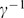, the fluctuation in 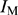 is effectively averaged out and has no significant effect on the fluctuation of 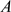. Thus, 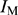 can be replaced by its average. Therefore, the reaction of the active state A is effectively reduced to be,
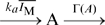
(10)which consists of the production reaction with the constant reaction rate 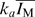 and the depletion reaction with rate 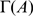. According to Ref. [16], the fluctuation strength of A at 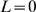 is given by 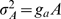, where 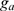 is the gain of 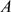 for the increase of the rate constant 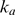, i.e., 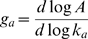. For scheme 10, the gain 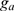 is given by the nonlinear degree 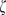, i.e. 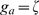. Thus, the fluctuation strength 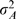 can be rewritten as 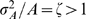, showing that the large fluctuation at 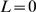 is due to the large gain 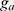, which is a result of the demethylation reaction with nonlinear rate 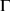. This scheme indicates the effective pathways with the strongest flux under the wild type condition. We should note that this reduced scheme does not necessarily mean that the chemoreceptor does not undergo reversible transitions between active and inactive states and not obey the detailed balance in the CheB and CheR deleted mutant cells without methylation and demethylation reactions.

For sufficiently large 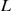, both the activation and inactivation reaction rates, 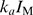 and 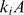, are much larger than the demethylation reaction rate 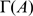, i.e., 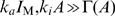. Thus, in this range of 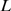, the demethylation reaction can be neglected. The number 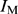 increases as the increase of 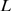, since the modification level increases as mentioned before. This indicates that for sufficiently large 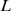 the fluctuation of 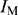 relative to its mean can be neglected due to its large concentration. It follows that the activation reaction rate is effectively constant. Therefore, the reaction of active state A is reduced to be:
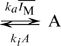
(11)where the distribution of 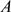 follows a Poisson distribution, which gives 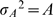. We also note that since the gain 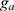 is unity for this scheme, the fluctuation intensity is given by 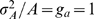. Thus, the decrease of the nonlinear demodification reaction rate relative to the activation and inactivation reaction rates is essential for the decrease of the fluctuation intensity of 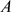 as the ligand concentration increases.

We should notice that the present result of nonadaptive fluctuation does not depend strongly on the several parameter values. As shown in Fig. 3D, even when the maximum rates 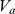 and 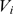 of activation and inativation reactions were increased or decreased ten times, the dependence of fluctuation on the ligand concentration was almost unchanged. Therefore, our result is applicable to the case when the transitions between active and inactive states are not fast processes and are considered to be away from equilibrium.

Note that the transition rates should be fast enough, otherwise 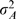 cannot decrease when the ligand concentration is high (Fig. 3D red line). We also studied the dependence of the nonadaptive property in the activity fluctuation on the ligand-independent inactivation reaction, which is characterized by 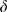 in 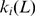. Such a ligand-independent inactivation reaction is expected for the case of bacterial chemotaxis. To obtain sufficiently strong response and to satisfy the bacterial chemotaxis (see below), 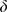 should be much smaller than 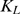 in 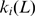. In the present case, we set 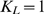. Thus, we have 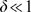. As shown in Fig. 3E, the nonadaptive property in the activity fluctuation is not strongly dependent on the parameter 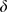.

In the present case, the variance 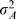 is a decreasing function of 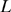 since 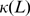 is an increasing function. This property of 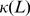 is required to satisfy the bacterial chemotaxis where the tumbling rate decreases when the ligand concentration increases. If 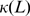 is a decreasing function of 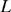, which is expected for a chemorepellent [8], the variance 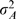 can be an increasing function. Therefore, the behavioral fluctuation of bacterial chemotaxis is dependent on the properties of the biochemical network.

**Figure 4:**
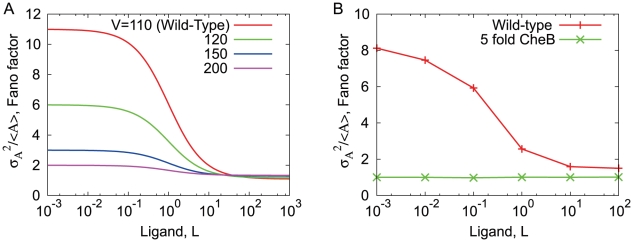
The fluctuation strength of the activity in our simple two-state model and the detailed model shown in **Fig. 2**. A: In our simple two-state model, the fluctuation strength of the activity and its ligand dependence decrease with the increase of 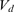. B: In the detailed model, an increase of CheB concentration results in a decrease of the fluctuation intensity of the activity and its ligand dependence.

The same result can be obtained in the detailed bacterial chemoreceptor model, in which multiple methylation sites are considered [24] (Fig. 2A). As shown in Fig. 2B, the stochastic time course of activity exhibits adaptive responses to the steplike changes in the chemoattractant concentration. However, the fluctuation intensity shown in Fig. 2C indicates clearly its ligand dependence, which is a decreasing function of the ligand concentration as is obtained in the simple two state model. Moreover, the parameter dependence of the fluctuation property is essentially the same in both models. From Eq. 4, the increase of 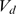 results in a decrease of the fluctuation intensity when 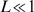 as shown in Fig. 4A. Since 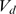 is the maximum reaction rate constant of the demodification reaction, it is equivalent to the increase of CheB concentration. Fig. 4B shows the ligand dependence of the fluctuation intensity in the detailed model. One finding, that an increase of CheB concentration results in a decrease of the ligand dependence and the reduction of the fluctuation intensity, shows good agreement with our theoretical result. Note that a five-fold increase of CheB concentration is sufficient for the fluctuation intensity to approach the Poissonian fluctuation, 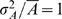. Therefore, our analysis extracts an essential feature of adaptive sensory systems, irrespective of the detailed aspects of the chemoreceptor reactions.

### Behavioral variability is dependent on ligand concentration

The above result indicates that bacterial behavior can exhibit a ligand dependence in its behavioral fluctuation, which is compatible with the property of adaptation. We study first the dependence of bacterial behavior on the chemoattractant concentration under spatially homogeneous conditions.

**Figure 5:**
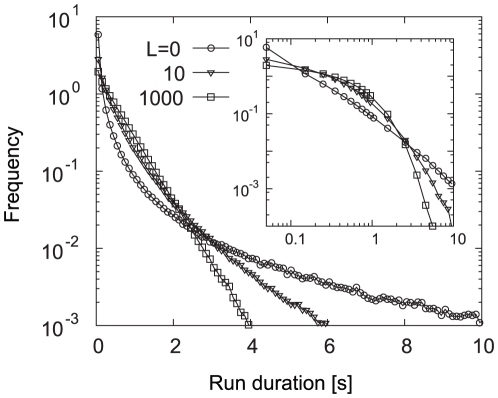
Probability distribution of run duration under the uniform background ligand concentrations. Run durations are measured for 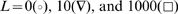. Inset: Logarithmic view of the identical plot.

Following the previous studies [25], [26], we extended our model to include motile machinery, the flagellar motor, which stochastically switches between the two states, “run” and “tumble” with transition rates 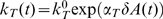 from run to tumble and 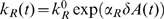 from tumble to run (see Materials and Methods). Here, 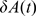 denotes the deviation of 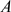 from the steady state value, and 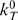, 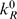, 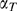 and 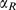 are constant parameters. Thus, the transition rates can be time-dependent. If the fluctuation of activity is small enough to be ignored, the transition rates 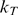 and 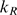 are constants and the run and tumble durations follow exponential distributions. This simple model can reproduce the switching behavior of the mutant cell, which expresses the constitutive active form of CheY [18]. For this mutant cell, the switching rates of rotation were decoupled from the receptor activity, showing that CCW duration, which is equivalent to the “run” state, was exponentially distributed. However, large fluctuations generated in the adaptation reaction can propagate to the motile machinery with ultrasensitivity [27], where it is amplified. Such fluctuations may affect the run and tumble duration distributions. In fact, when the chemoattractant is absent, the run duration exhibits a heavy-tailed distribution, where the probability to have a longer run duration is not bounded by an exponential distribution (Fig. 5 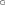) [18], [26]. Correlated with the decrease in the fluctuation of activity as the ligand concentration increases (Fig. 3C), such behavioral fluctuation is reduced and approaches an exponential distribution at high chemoattractant concentration (Fig. 5 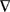 and 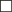). Thus, the run duration is changed from a heavy-tailed distribution to an exponential one as the chemoattractant concentration increases.

The distribution of durations of counterclockwise (CCW) and clockwise (CW) rotations has been measured in the absence of chemoattractant [18]. The CCW and CW rotations correspond to run and tumble, respectively. The duration of CCW rotation was found to obey the heavy-tailed distribution, whereas CW duration was distributed exponentially. This experiment suggested that the temporal fluctuation generated at the chemoreceptor propagates to the motor, leading to the run duration being distorted from an exponential distribution. We also note that the earlier experiment by Block et al. demonstrated that in the presence of chemoattractant, the CCW duration was distributed exponentially [10]. The amount of ligand in their experiment was comparable to the dissociation constant of chemoreceptor. Our result above could consistently explain the apparent discrepancy between the two experiments, by considering the dependences of fluctuation on the ligand concentration.

### Chemotactic performance can be enhanced by fluctuations

Such dependence of the run length distribution on the ligand concentration would enable the bacterial motility to depend on the chemoattractant level, even though the sensory system exhibits the property of perfect adaptation.

**Figure 6:**
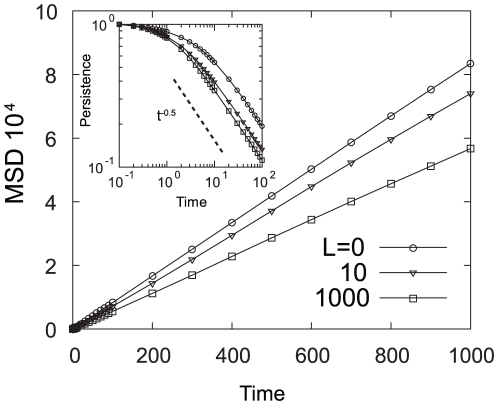
Mean Square Displacements for the bacterial motility. MSDs are plotted as functions of time 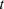 for 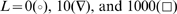. At the long time scale, 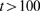, bacterial motion is regarded as a random walk, with linear dependence on time. Inset: Persistence of the bacterial motion plotted as functions of time 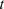. The persistence is unity for a short time, indicating that the motion is ballistic, then, it decays in proportion to 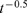. The broken line is proportional to 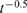.

In particular, the heavy-tailed distribution of run length could give rise to a motility spreading in an area larger than the motion of an ordinary random walk. Thus, higher mobility would be expected when the environmental ligand concentration is low. The mobility of bacteria in a uniform chemoattractant concentration can be characterized by the mean square displacement (MSD) (Fig. 6), calculated as 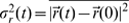, where 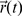 is the position at time 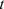. The MSD 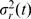 is the variance of the distribution of bacteria at time 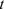 starting from the same position at 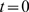, which increases linearly for sufficiently long time scales 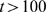 as 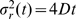, where 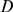 is the effective diffusion constant. The result shows that 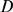 is larger in the absence of ligand and decreases as 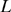 increases. Such a dependence of motility on the ligand concentration is a consequence of the fluctuation in the adaptation reaction that is dependent on the chemoattractant concentration. Considering a “noiseless cell” in which the activity 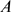 does not contain intrinsic fluctuations, the effective diffusion constant 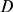 of such a noiseless cell is constant without dependence on 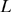. The high motility of the wild-type cells in a low concentration regime is also seen in the directional persistence of cell migration, 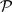, defined as the ratio between the net displacement equivalent to 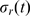 and the total length of the motional trajectory in an interval 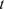. The persistence 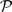 is a decreasing function of time 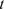. As shown in Fig. 6 inset, the more ligand concentration increases, the faster persistence 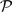 falls with time. Therefore, for low ligand concentrations, the bacteria can spread into a wider area within a short time scale.

**Figure 7:**
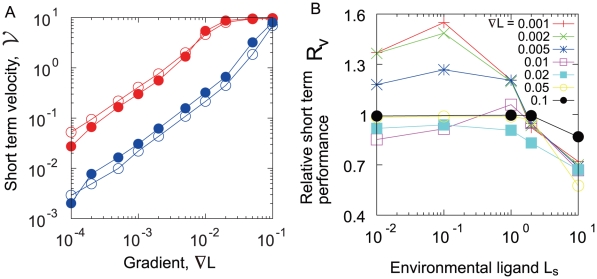
Short term velocity along the chemical gradient. A. Relationship between short term velocity and the gradient. Open circles represent 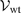 for the wild-type, and closed circles for the noiseless cell, 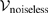. 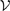 is measured for 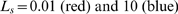. B. 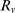, the normalized velocity of 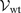 by 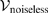, is plotted against the 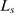, the ligand concentration at the starting point.

Under the chemoattractant gradient a bacterium exhibits a directional motion, which can be quantified by the net velocity 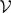 for relatively short periods of time [28], [29] (see Materials and Methods). 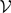 shows a linear dependence on the steepness of chemoattractant gradient (Fig. 7A). For low background concentration, the velocity of the wild-type cell 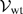 is larger than that of the noiseless cell, 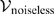, indicating that the fluctuation improves the chemotactic performance. For a high background concentration, 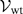 is smaller than 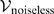. The ratio 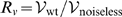 shown in Fig. 7B indicates that the enhancement of chemotactic performance is prominent when the background concentration is low and the gradient is shallow. While the fluctuation in the sensory apparatus may disturb the ability of gradient sensing as noise, our result reveals the opposite role in chemotaxis. Particularly in the low concentration regime, bacteria search for a chemoattractant in a wider area. Once they reach a shallow gradient, they climb up quickly.

**Figure 8:**
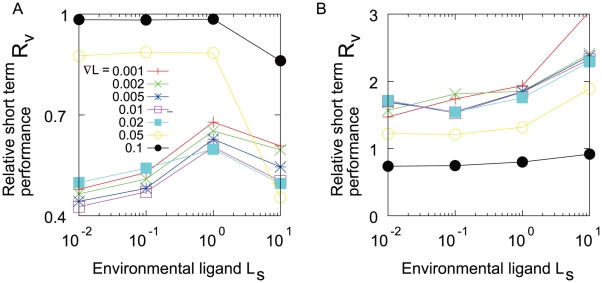
The ratio 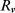 between the velocities of mutant I (A) and mutant II (B) and their noiseless cells plotted as functions of ligand concentration 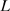.

To clarify the reason for this increase, we investigated several mutants. We first studied mutant **I**, which has the modification and demodification rates, 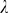 and 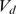, that are 10 times faster than those rates of the wild-type cell. The activity 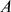 of this mutant has a correlation time of fluctuation that is faster than that of the wild-type cell. In mutant **I**, the fluctuation intensities of the switching rates of the motility, 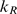 and 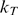, become smaller, resulting in the disappearance of the tail in the run length distribution despite the large activity fluctuation. To study the effect of the stochastic fluctuation of activity, we also investigated the noiseless cell of the mutant **I**. In Fig. 8A, we plot the ratio between the velocity 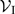 of the mutant **I** and the velocity 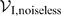 of its noiseless cell, i.e., 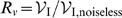. The ratio is less than unity, indicating that the performance is not improved by the stochastic fluctuation of 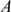 in mutant **I**. Next we studied another mutant **II**, in which the inactivation pathway is deleted (see Fig. 3C green). Mutant **II** shows a large fluctuation and a heavy-tailed distribution of run length irrespective of the environmental ligand concentration. We also investigated the corresponding noiseless mutant. As shown in Fig. 8B, the ratio between the velocities of mutant **II**, 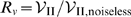, is larger than that of the wild-type cell for any background concentration of 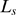 unless the ligand gradient is quite steep 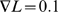. These results indicate that the increase in the velocity 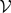 is mainly the consequence of the heavy-tailed distribution of run length.

**Figure 9:**
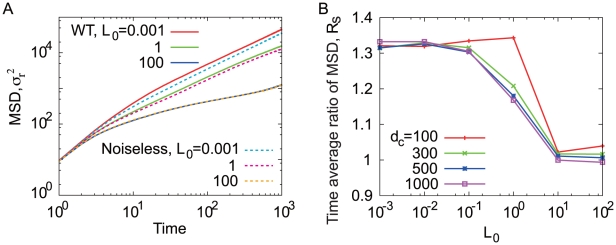
Mobility from the chemoattractant source. A: The mean square displacement, 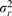, in the presence of the chemical gradient, 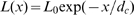. 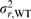 are shown by solid lines, and 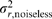 by broken lines. The individual bacterium starts to swim in a one dimensional field from the higher end of the gradient, 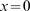. B: Time averaged ratio 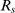 against the background concentration for various value of steepness. MSDs are averaged for 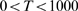, which is long enough to capture the bacterial long term behavior.

Bacteria spread with time, even from a source of chemoattractant, because of the biased random walk. To quantify the degree of spreading with time, we measured the temporal change of 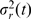 for the bacterial population put on the tip of the exponential gradient for various value of steepness and concentrations at the tip. To compare 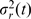 of the wild-type, 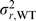, with that of the noiseless cell, 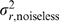, we introduce their time averaged ratio 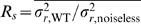. As shown in Fig. 9B, 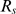 is a decreasing function of the background concentration. Therefore, whereas the fluctuation of the adaptation reaction enhances the spread of bacteria from an area of low chemoattractant concentration, in a high concentration area the spreading to weaken aggregation is not increased.

The above results indicate that the stochastic fluctuation of the sensory system does not reduce the chemotactic performance in most situations. In particular, under the low background concentration, the large fluctuation of the sensory system leads to increasing the cell motility and chemotactic speed. We should note that to study the increase of the chemotactic performance, the cell with stochastic sensory system (wild type) was compared with the cell with the sensory system without stochasticity (noseless cell). The time constants and the response sensitivity are the same between these two types of cells. Thus, the increase in the chemotactic performance is purely the consequence of the effect of noise, but is not the effect of the increase in time constant as in the case of Emonet and Cluzel [19]. When reaching a high concentration area, bacteria suppress spreading by decreasing the stochastic fluctuations of the chemoreceptor circuit. Consequently, a bacterial population achieves higher aggregation performance toward the chemoattractant by switching its behavior depending on the chemoattractant concentration.

### Conclusion

**Figure 10:**
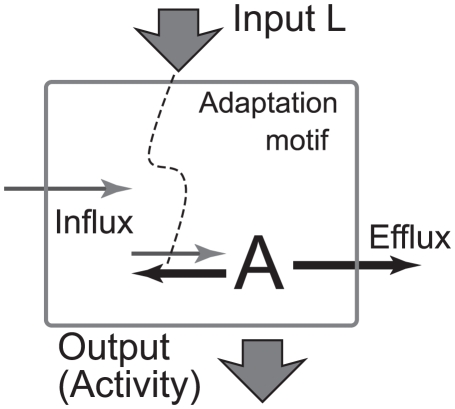
Schematic diagram of the adaptation motif with nonadaptive fluctuations. The motif consists of a set of chemical species. In the figure, only molecule A is shown. All the reaction paths are drawn in thick line. The output is the number of molecules of A, which is activity of the motif. The input signal modulates the activity through the activation and inactivation reaction rates. The influx is the supply reaction for the motif, whereas the efflux is the depletion reactions from it. The efflux reaction is typically the enzymatic reaction, which generate a larger fluctuation. When the efflux is dependent only on the activity, and both the influx and efflux are independent of other molecules, the activity exhibits a perfect adaptation at steady state. For the nonadaptive fluctuation, at least two depletion pathways are necessary for molecule A. One pathway in the motif should be dependent on the input, and one must be a nonlinear reaction.

In the present paper, we studied the stochastic nature of the adaptive sensory systems, such as ion channels, and membrane receptors. While the activity shows adaptation on average, its temporal fluctuation is a nonadaptive property, which is sensitive to the environmental ligand concentration. The ligand dependence is revealed when there exist two depletion pathway of the activity, one of which exhibits a non-first order property (Fig. 10). Since our analysis is performed in a simple prototypic model, the nonadaptive fluctuation is a property common to the adaptive systems studied here. In the present paper, we further analyzed the bacterial chemoreceptor, which is the best studied adaptive sensory system. We have shown that the nonadaptive fluctuation influences the motile property through the switching reaction of the flagellar motor, resulting in the behavioral fluctuation being dependent on the background ligand concentration. The ligand dependence of bacterial behavior influences cell motility under uniform environment, which increases the chemotactic performance. Therefore, the nonadaptive fluctuation can carry information of the environmental ligand concentration. By altering the behavior depending on the fluctuation intensity of the sensory system, the cell can adaptively change its behavior to suit the environmental conditions. Our result indicates a possible function of stochastic fluctuation in that it can transmit information downstream, even though this cannot be done by its average.

## Materials and Methods

### Derivation of fluctuation strength in the two state model

To derive Eq. (4), we consider the chemical Langevin equation for scheme 2, given by Eq. (3). The concentrations 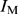 and 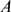 can be written as
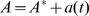
(12)

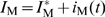
(13)where 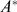 and 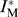 are the steady state solution without noise, and 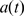 and 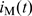 are the time dependent deviations about 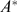 and 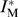. To obtain the variance 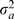 of 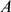, we performed the linear noise approximation, which gives the equation,
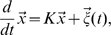
(14)where 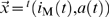, 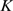 is the linear regression matrix given by
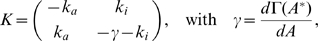
(15)and 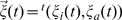 is the white Gaussian noise with zero mean and 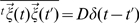 with
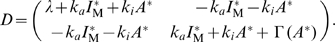
(16)Using the relation 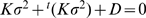 with the covariance matrix 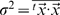, we obtain
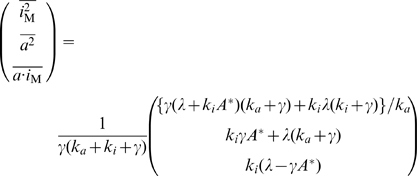
(17)Introducing new variables, 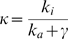 and 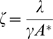, we obtain Eq. (4).

### Fluctuation strength in the reduced activity dependent kinetic model

The linearized equation of Eq. (7) with linear noise approximation is
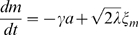
(18)where, 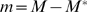. Under the equilibrium condition between 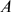 and 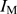, the fluctuation strength of 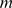, 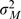, is given,
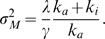
(19)With Eq. (8), we have Eq. (9).

### Simulation of the receptor sensory reactions

The stochastic simulation of scheme 2 is performed by *τ*-leap algorithm [30], which approximately simulates the stochastic dynamics of the chemical reactions in discrete time steps. For the numerical simulation of noiseless cell, we calculated the ordinary differential equations, where the noise terms are omitted in Eq. (3), with the Euler method.

The response and adaptation times of the sensory system of bacterial chemotaxis are respectively 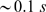 and 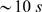 for a small step increase of chemoattractant [8]. We determined the reaction parameters to reproduce these time constants. In the model given by scheme 2, the response time 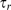 and adaptation time 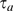 are approximately given by
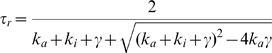
(20)

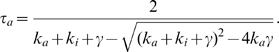
(21)In the case of 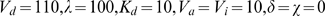, and 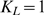, we obtained 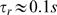 and 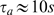 for the low background 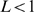, which is consistent with the above time constants.

### The effect of nonlinearity on average activity

We notice that the statistical average 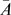 is larger than the solution of the kinetic equation without the noise term, 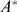, particularly for a low background ligand concentration, as shown in Fig. 3B. The deviation is also found in the fluctuation intensity as shown in Fig. 3C, where the estimated variance given in Eq. (4) is smaller than the variance obtained by numerical simulations. These deviations are due to the nonlinearity of the demodification reaction. From Eq. (12), the statistical average of 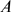 at steady state is given by 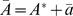 with 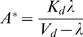. The average 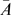 can be obtained by solving the equation,
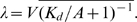
(22)By performing a Taylor expansion at 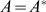 up to the second order of 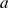, we have
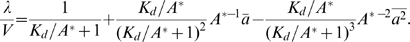
(23)Note that 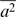 increases in proportion to the increase of 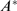. Thus, we expand 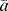 and 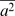 as a series of 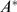 as 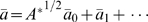 and 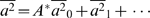. By equating the terms in each power of 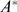 in Eq. (23), we have 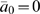, and 
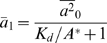
. Thus, the steady state value up to the zeroth order of 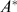 is given by,
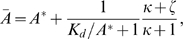
(24)which shows good agreement with the numerical result shown in Fig. 3B.

### Model of bacterial motility

The motion of a bacterium consists of “run” and “tumble”. Between these two states, we consider stochastic transitions as
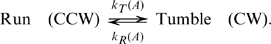
(25)where 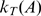 and 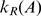 are the activity-dependent switching rates as shown in the text [25], [26]. The parameter values that we used in the simulation were 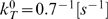, 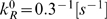, 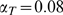, and 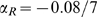. The combination of 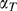 and 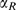 determines the dependence of the fraction of CW state in time on the activity 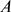. The parameter values of 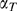 and 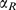 were chosen to reproduce the reported experimental result [27]. The value of 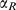 was chosen to have an exponential distribution for the tumbling duration even in the absence of ligand, as was reported in Ref. [18]. For each state of “run” and “tumble” motions, bacteria are considered to show a rotational Brownian motion with respective constant speeds, 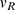 and 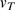. The direction of motion in a two dimensional space, 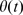, follows a stochastic differential equation given by 
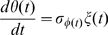
, where 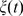 is a white Gaussian noise with 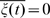 and 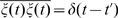, and 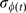 is the strength of noise with 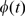 indicating the state of motion, i.e., 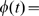 “r” and “t” for “run” and “tumble” motions, respectively. In the present paper, we use 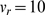, 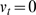, 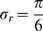 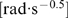, and 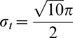 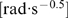. For the stochastic simulation of bacterial motility, we used the *τ*-leap algorithm for the receptor reactions and motor switching, and the Euler-Maruyama method for the bacterial movement in the same discrete time step.

### Measurement of chemotactic performance

The short term velocity, 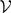, under the chemoattractant gradient [28] is given by
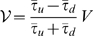
(26)where 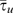 represents the mean duration of successive run and tumble intervals for upward motion, starting at a given position and 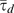 for downward motion. Multiplying by the swimming velocity 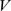, 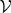 represents the averaged upward velocity along the chemical gradient between two tumbling motions. 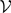 is measured in the presence of the linear gradient, 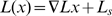, where 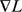, 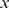 and 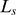 denote the chemical gradient, the position of a bacterium, and the ligand concentration at starting point, 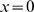. At 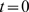, cells are adapted to the ligand concentration, 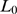, and start to move along or against the gradient.

To obtain 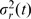 in the presence of the chemical gradient, the bacterial population is placed at the top of the gradient. As the initial condition, the receptor activity reaches the steady state at the concentration of the top of the gradient. 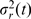 is calculated from the bacterial population, which consists of 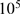 cells. There exists a reflective wall at 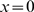, prohibiting the bacterium from going across the boundary 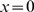. This boundary condition is equivalent to the ligand distribution spreading exponentially on both positive and negative sides of 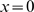.

## References

[1] Koshland DE, Goldbeter A, Stock JB (1982). Amplification and adaptation in regulatory and sensory systems.. Science.

[2] Barkai N, Leibler S (1997). Robustness in simple biochemical networks.. Nature.

[3] Friedlander T, Brenner N (2009). Adaptive response by state-dependent inactivation.. Proceedings of the National Academy of Sciences.

[4] Marom S, Levitan I (1994). State-dependent inactivation of the Kv3 potassium channel.. Biophysical journal.

[5] Ferguson S, Caron M (1998). G protein-coupled receptor adaptation mechanisms.. Seminars in Cell and Developmental Biology.

[6] Berg H, Brown D (1972). Chemotaxis in escherichia coli analysed by three-dimensional tracking.. Nature.

[7] Macnab R, Koshland (1972). The gradient-sensing mechanism in bacterial chemotaxis.. Proc Natl Acad Sci U S A.

[8] Block S, Segall J, Berg H (1982). Impulse responses in bacterial chemotaxis.. Cell.

[9] Sourjik V, Berg H (2002). Receptor sensitivity in bacterial chemotaxis.. Proc Natl Acad Sci U S A.

[10] Block S, Segall J, Berg H (1983). Adaptation kinetics in bacterial chemotaxis.. J Bacteriol.

[11] Asakura S, Honda H (1984). Two-state model for bacterial chemoreceptor proteins. the role of multiple methylation.. J Mol Biol.

[12] Rao C, Kirby J, Arkin A (2004). Design and diversity in bacterial chemotaxis: a comparative study in Escherichia coli and Bacillus subtilis.. PLoS biology.

[13] Kollmann M, Løvdok L, Bartholomé K, Timmer J, Sourjik V (2005). Design principles of a bacterial signalling network.. Nature.

[14] Hansen C, Endres R, Wingreen N (2008). Chemotaxis in Escherichia coli: a molecular model for robust precise adaptation.. PLoS Comput Biol.

[15] Rao C, Wolf D, Arkin A (2002). Control, exploitation and tolerance of intracellular noise.. Nature.

[16] Shibata T, Fujimoto K (2005). Noisy signal amplification in ultrasensitive signal transduction.. Proc Natl Acad Sci U S A.

[17] Tostevin F, Ten Wolde P (2009). Mutual information between input and output trajectories of biochemical networks.. Physical Review Letters.

[18] Korobkova E, Emonet T, Vilar J, Shimizu T, Cluzel P (2004). From molecular noise to behavioural variability in a single bacterium.. Nature.

[19] Emonet T, Cluzel P (2008). Relationship between cellular response and behavioral variability in bacterial chemotaxis.. Proc Natl Acad Sci U S A.

[20] Goldbeter A, Koshland JDE (1981). An amplified sensitivity arising from covalent modification in biological systems.. Proc Natl Acad Sci U S A.

[21] Gillespie D (2000). The chemical Langevin equation.. The Journal of Chemical Physics.

[22] Tu Y, Berg H (2008). Modeling the chemotactic response of escherichia coli to time-varying stimuli.. Proc Natl Acad Sci U S A.

[23] Borkovich K, Alex L, Simon M (1992). Attenuation of sensory receptor signaling by covalent modification.. Proc Natl Acad Sci U S A.

[24] Barkai N, Alon U, Leibler S (2001). Robust amplification in adaptive signal transduction networks.. Comptes Rendus de l'Academie des Sciences Series IV Physics.

[25] Khan S, Macnab R (1980). The steady-state counterclockwise/clockwise ratio of bacterial flagellar motors is regulated by protonmotive force.. J Mol Biol.

[26] Tu Y, Grinstein G (2005). How white noise generates power-law switching in bacterial flagellar motors.. Phys Rev Lett.

[27] Cluzel P, Surette M, Leibler S (2000). An ultrasensitive bacterial motor revealed by monitoring signaling proteins in single cells.. Science.

[28] de Gennes P (2004). Chemotaxis: the role of internal delays.. Eur Biophys J.

[29] Clark D, Grant L (2005). The bacterial chemotactic response reflects a compromise between transient and steady-state behavior.. Proc Natl Acad Sci U S A.

[30] Gillespie D (2001). Approximate accelerated stochastic simulation of chemically reacting systems.. The Journal of Chemical Physics.

[31] Gillespie D (1977). Exact stochastic simulation of coupled chemical reactions.. The journal of physical chemistry.

